# MicroRNAs, T follicular helper cells and inflammaging

**DOI:** 10.18632/oncotarget.6025

**Published:** 2015-10-07

**Authors:** Jason E. Hawkes, Ryan M. O'Connell

**Affiliations:** Department of Dermatology and Department of Pathology, Division of Microbiology and Immunology, University of Utah, Salt Lake City, UT, USA

**Keywords:** microRNAs, inflammation, T cells

Advanced age is associated with a declining ability to mount satisfactory immune responses (i.e. immunosenescence) resulting in increased infection susceptibility and diminished vaccine-elicited immune responses. Paradoxically, aged individuals also have a reduced ability to resolve certain types of inflammation (i.e. inflammaging) leading to chronic, low-grade inflammatory responses and the development of inflammation-associated comorbidities, such as cardiovascular disease, autoimmunity, and malignancy. The contribution of immunosenescence and inflammaging to human disease is substantial, thus highlighting the importance of understanding the mechanisms responsible for these processes.

Recently, T follicular helper (Tfh) cells have been implicated in the regulation of chronic inflammation, and our recent study suggests that this may also include inflammaging [1]. Tfh cells make up a heterogeneous group of CD4^+^ T cells with a diverse set of functions, principally providing help for the B cell response. Tfh cells also contribute to the selection of GC B cells, tumor microenvironment, and regulation of commensal microbiota [2]. Bcl6, STAT3, IRF4, BATF, Ascl2, and c-MAF are among the key transcription factors involved in the complex, multistage differentiation process of Tfh cells [3, 4]. Despite a flurry of new information about the multifaceted roles of Tfh cells and molecular mechanisms that regulate their biology, additional gaps exist in our understanding of their differentiation and their roles in inflammatory conditions. Of relevance, the study of non-coding RNAs is beginning to offer additional insights about the differentiation of Tfh cells and their contribution to inflammaging.

More than 70% of the human genome is transcribed into non-coding RNAs, which include microRNAs [5]. These endogenous, small RNAs exert their effect through the post-transcriptional modification of messenger RNA and are essential for an array of normal and aberrant biological processes [6]. miR-146a and miR-155 are among the microRNAs shown to be key mediators of the immune response. miR-155 is a potent pro-inflammatory enhancer that is induced by a variety of Toll-like receptor ligands, cytokines, and antigens, whereas miR-146a functions as a safeguard against autoimmunity and promotes resolution of the inflammatory response by limiting activation of T cells [7]. *miR-146a^−/−^* mice undergo an age-dependent decrease in life expectancy due to severe immune dysfunction and the development of hematopoietic tumors, malignancy, and inflammation within secondary lymphoid organs. The early activation of CD4^+^ T cells in the hematopoietic compartment is fundamental to this systemic inflammatory process. Thus, *miR-146a^−/−^* mice provide investigators with a remarkable model of low-grade, chronic inflammation and the unique opportunity to study inflammaging.

We recently described the regulatory roles of miR-155 and miR-146a during the differentiation of Tfh cells and the development of chronic inflammation using the miR-146a-deficient mouse model [1]. In order to study the role of miR-155 on inflammaging, we aged wild-type (WT), *miR-146a^−/−^*, and *miR-146a^−/−^miR-155^−/−^* (DKO) mice for 7–10 months. We found that the tissues of middle-aged *miR-146a^−/−^* mice were associated with increased numbers of activated CD4^+^ T cells, spontaneous GC formation, and the development of circulating dsDNA autoantibodies [1]. The CD4^+^ T cell population in these mice also expressed higher levels of miR-155 compared to WT controls and were enriched for Tfh cells along with Tfh-related cytokines (e.g. IL-21) and genes (e.g. Bcl6, Cxcr5, Pd1, and Icos) [1]. Enhanced expression of miR-155 was not observed in the B cell compartment. Importantly, WT and DKO mice had similar phenotypes and did not share the pro-inflammatory features of the *miR-146a^−/−^* mice. This suggests that miR-155 is required for the accumulation of activated CD4^+^ T and Tfh cells, increased GC formation, and the production of autoantibodies in aged miR-146a-deficient mice [1]. This notion is further supported by the observation that clustering analysis of gene expression in CD4^+^ T cells from DKO mice was most comparable to the expression pattern for *miR-155^−/−^* mice [1].

Next, we sought out to determine whether the regulatory effect of miR-155 on the accumulation of Tfh cells and the GC response was intrinsic or extrinsic to CD4^+^ T cells in miR-146a-deficient mice. In order to test this, we created T-cell specific miR-155 conditional knockout (*Cd4-cre Mir155^fl/fl^*) mice, where the miR-155 gene is floxed and Cre is driven by the CD4 locus. CD4^+^ T cells lacking miR-155 also displayed impaired Tfh cell formation and a reduction in their ability to produce antigen-specific antibodies. We also found that the spontaneous, age-dependent accumulation of Tfh and GC B cells in the spleen and lymph nodes of *miR-146a^−/−^* mice was dependent on the expression of miR-155 in T cells upon analyzing *miR-146a^−/−^ Cd4-cre Mir155^fl/fl^* mice [1]. These observations are due in part to the inhibition of multiple miR-155 target genes, including the knockdown of two novel targets: Fosl2 and Peli1 [1].

This study expands our current understanding of the role of microRNAs in the process of Tfh differentiation, and also describes how this process may promote inflammaging. However, significant research gaps still exist regarding the role of Tfh cells in other aspects of human disease and the details of their regulatory network during various stages of development and function. It also remains to be seen whether the molecular pathways and regulatory networks controlling Tfh cell development and inflammaging in murine models are truly representative of those in humans. Future studies are necessary to answer these important questions.

As the elderly population grows worldwide, the mechanisms of age-related immune dysfunction and the burden of chronic inflammatory conditions will be of ever-increasing importance. Understanding how microRNAs and Tfh cells contribute to inflammation and aging is an exciting area of research. Additionally, the development of new therapies aimed at the targeting of specific microRNAs and/or the regulatory mechanisms of Tfh cell differentiation could represent a possible strategy to augment the immune response and offset the deleterious effects of inflammaging.
